# Long-acting lenacapavir protects macaques against intravenous challenge with simian-tropic HIV

**DOI:** 10.1016/j.ebiom.2023.104764

**Published:** 2023-08-23

**Authors:** Adrienne E. Swanstrom, Robert J. Gorelick, Jorden L. Welker, Fabian Schmidt, Bing Lu, Kelly Wang, William Rowe, Matthew W. Breed, Kristin E. Killoran, Joshua A. Kramer, Duncan Donohue, James D. Roser, Paul D. Bieniasz, Theodora Hatziioannou, Cathi Pyle, James A. Thomas, Charles M. Trubey, Jim Zheng, Wade Blair, Stephen R. Yant, Jeffrey D. Lifson, Gregory Q. Del Prete

**Affiliations:** a AIDS and Cancer Virus Program, Frederick National Laboratory for Cancer Research, Frederick, MD, USA; b Gilead Sciences, Foster City, CA, USA; c Laboratory Animal Sciences Program, Frederick National Laboratory for Cancer Research, Frederick, MD, USA; d DMS Applies Information Management Sciences, Frederick National Laboratory for Cancer Research, Frederick, MD, USA; e Laboratory of Retrovirology, Rockefeller University, New York, NY, USA

**Keywords:** HIV, Pre-exposure prophylaxis, PrEP, Nonhuman primate, Macaque, Capsid

## Abstract

**Background:**

Long-acting subcutaneous lenacapavir (LEN), a first-in-class HIV capsid inhibitor approved by the US FDA for the treatment of multidrug-resistant HIV-1 with twice yearly dosing, is under investigation for HIV-1 pre-exposure prophylaxis (PrEP). We previously derived a simian-tropic HIV-1 clone (stHIV-A19) that encodes an HIV-1 capsid and replicates to high titres in pigtail macaques (PTM), resulting in a nonhuman primate model well-suited for evaluating LEN PrEP in vivo.

**Methods:**

Lenacapavir potency against stHIV-A19 in PTM peripheral blood mononuclear cells in vitro was determined and subcutaneous LEN pharmacokinetics were evaluated in naïve PTMs in vivo. To evaluate the protective efficacy of LEN PrEP, naïve PTMs received either a single subcutaneous injection of LEN (25 mg/kg, N = 3) or vehicle (N = 4) 30 days before a high-dose intravenous challenge with stHIV-A19, or 7 daily subcutaneous injections of a 3-drug control PrEP regimen starting 3 days before stHIV-A19 challenge (N = 3).

**Findings:**

In vitro, LEN showed potent antiviral activity against stHIV-A19, comparable to its potency against HIV-1. In vivo, subcutaneous LEN displayed sustained plasma drug exposures in PTMs. Following stHIV-A19 challenge, while all vehicle control animals became productively infected, all LEN and 3-drug control PrEP animals were protected from infection.

**Interpretation:**

These findings highlight the utility of the stHIV-A19/PTM model and support the clinical development of long-acting LEN for PrEP in humans.

**Funding:**

10.13039/100005564Gilead Sciences as part of a Cooperative Research and Development Agreement between 10.13039/100005564Gilead Sciences and 10.13039/100012728Frederick National Lab; federal funds from the 10.13039/100000054National Cancer Institute, 10.13039/100000054National Institutes of Health, under Contract No. 75N91019D00024/HHSN261201500003I; 10.13039/100000002NIH grant R01AI078788.

**Research in context**

**Evidence before this study**

When taken as prescribed, daily oral pre-exposure prophylaxis (PrEP) is highly effective for the prevention of HIV-1 transmission. However, barriers to daily pill-taking limit the overall efficacy of daily oral PrEP. The use of long-acting agents for PrEP that can accommodate less-frequent dosing represents a promising alternative approach that may overcome some of the limitations of daily oral regimens. Two recent clinical trials (HPTN 083 and HPTN 084) comparing daily oral PrEP with long-acting cabotegravir PrEP, administered once every eight weeks via intramuscular injection, demonstrated the potential benefit of long-acting drugs. In both trials, long-acting cabotegravir showed significantly superior protection against HIV-1 acquisition compared with daily oral regimens, likely due to regimen non-adherence in a portion of the daily oral PrEP group participants. Lenacapavir (LEN) is a potent, selective inhibitor of HIV-1 capsid function that has been formulated for prolonged plasma exposures following subcutaneous administration. It was recently approved for the treatment of multidrug resistant HIV-1 with dosing as infrequent as twice per year. Lenacapavir’s unique mechanism-of-action, which distinguishes it from all other approved antiretroviral drugs, combined with its potency and pharmacologic properties make it attractive as a potential new long-acting PrEP agent.

**Added value of this study**

To evaluate LEN PrEP in a relevant nonhuman primate model, we utilized a simian-tropic HIV-1 (stHIV-A19) that contains an HIV-1 capsid protein and can replicate to high levels in pigtail macaques (PTMs). We demonstrate the relevance of this model for LEN evaluation by first showing that, unlike simian immunodeficiency virus (SIV) and SIV-based viruses, the sensitivity of stHIV-A19 to LEN is comparable to the sensitivity of HIV-1 to LEN. We also show that following subcutaneous injection, LEN demonstrates prolonged plasma exposures in PTMs in vivo. Finally, in a high-dose, intravenous challenge experiment, we show that LEN prevents infection in PTMs challenged with stHIV-A19, with no evidence of virus emergence upon LEN washout or subsequent experimental CD8 depletion intended to reveal any potential undetected infections.

**Implications of all the available evidence**

Our findings, combined with emerging clinical evaluations of LEN, support the development of LEN for PrEP, including in people who inject drugs.

## Introduction

Despite decades of concerted public health efforts to reduce HIV-1 transmissions, there were an estimated 1.5 million new HIV-1 infections worldwide in 2021.^1^ Pre-exposure prophylaxis (PrEP), the use of antiretroviral drugs (ARVs) such as oral emtricitabine/tenofovir disoproxil fumarate (FTC/TDF) or emtricitabine/tenofovir alafenamide (FTC/TAF) in uninfected individuals, can be highly effective for preventing new infections.^2^, ^3^, ^4^, ^5^, ^6^, ^7^, ^8^, ^9^, ^10^ However, the efficacy of daily oral PrEP has been limited by its dependence on strict drug regimen adherence and the attendant maintenance of protective drug concentrations in PrEP recipients.^2^^,^^3^^,^^8^^,^^9^^,^^11^, ^12^, ^13^ For instance, in the iPrEX trial, which evaluated daily oral PrEP with FTC/TDF, there was only a modest 44% lower incidence of HIV-1 infection overall in the PrEP group compared with placebo controls overall, but when the analysis was focused only on those PrEP group participants with detectable intracellular drug levels, indicative of regimen adherence, there was a 92% reduction in HIV-1 infection risk.^2^

Antiretrovirals with long-acting pharmacokinetic (PK) properties that can accommodate less frequent drug administration represent a promising PrEP strategy that may overcome the limitations of agents that require daily dosing. The potential effectiveness of long-acting PrEP was recently demonstrated in two clinical trials that found PrEP with a long-acting formulation of cabotegravir (CAB-LA), an integrase strand-transfer inhibitor administered via intramuscular injection once every 2 months, was significantly more effective than daily oral PrEP at preventing HIV acquisition.^14^^,^^15^ Based on these studies, CAB-LA was recently granted FDA approval for PrEP. Although CAB-LA represents a new alternative option to daily oral PrEP regimens, it must be administered intramuscularly by a health care provider every 8 weeks, and its consistent use may therefore be burdensome for some people who could benefit from PrEP. Thus, there remains an unmet clinical need for additional long-acting PrEP options to address HIV-1 transmission globally.

Lenacapavir (LEN, formerly GS-6207) is a potent and selective capsid protein (CA) targeted inhibitor that has been formulated for sustained plasma drug exposures. When administered as monotherapy to people with HIV-1, a long-acting LEN formulation suppressed HIV-1 replication, with >100-fold maximum reductions in plasma viral loads after 9 days of treatment in study participants that received 450 mg of the drug.^16^ In a Phase 1b clinical trial, a single subcutaneous (SC) injection of 900 mg LEN resulted in mean plasma drug concentrations that were maintained at ≥6-fold above the drug’s plasma protein binding-adjusted 95% effective concentration (PA-EC_95_) for 6 months after administration.^17^ In a recent Phase 3 clinical trial, LEN demonstrated good safety and antiviral efficacy with twice-yearly SC dosing in people with multidrug resistant HIV-1 infection,^18^ findings that led to regulatory approval in the US, EU, UK, and Canada for use in combination with other ARVs to treat multidrug-resistant HIV-1 infection. In addition to twice-yearly SC dosing potential, LEN has several features that make it attractive as a potential new long-acting agent for HIV-1 PrEP. It has picomolar potency against each of the major HIV-1 subtypes and, because it targets a viral protein other than those targeted by any currently approved antiretroviral drug, it retains its potency against viral variants with resistance to other drug classes.^16^^,^^19^ Conversely, resistance to LEN is not expected to confer resistance to other drug classes, as indicated by in vitro experiments utilizing representative agents from other drug classes.^16^^,^^20^

In preclinical studies, a long-acting formulation of a structural analogue of LEN, called GS-CA1, showed efficacy as monotherapy against HIV-1 in a humanized mouse model of HIV-1 infection.^20^ Recently, GS-CA1 was evaluated for its capacity to protect rhesus macaques against repeated intrarectal challenges with chimeric simian-human immunodeficiency virus (SHIV).^21^ Following a single SC injection with GS-CA1 or placebo, study animals were intrarectally challenged weekly with SHIV. While all placebo control animals became infected within 15 or fewer weekly challenges, none of the animals that received GS-CA1 became infected until challenge 16 or later, when GS-CA1 plasma concentrations declined to less than twice the GS-CA1 PA-EC_95_.^21^

Given the reduced potency of LEN against HIV-2^16^ and viruses containing an SIV CA^21^ compared with circulating HIV-1 variants, we sought to evaluate LEN PrEP in a nonhuman primate (NHP) model that utilizes a virus with an authentic HIV-1 CA. We previously derived a minimally-chimeric HIV-1 infectious molecular clone, termed simian-tropic HIV-1 clone A19 (stHIV-A19), that can replicate to high levels in vivo in pigtail macaques (PTMs), which in contrast to rhesus macaques do not restrict HIV-1 replication via capsid-targeted TRIM5 proteins.^22^^,^^23^ Derivation of stHIV-A19 involved replacement of the *vif* gene in the HIV-1 genome with an SIV *vif* gene, enabling the virus to overcome host-specific restriction by macaque apoliprotein B mRNA editing enzyme catalytic subunit 3 (APOBEC3) antiviral proteins, followed by serial passage in PTMs and subsequent molecular cloning of a host-adapted variant.^22^^,^^23^ While the resultant virus requires experimental CD8 depletion of the PTM host at the time of inoculation for sustained elevated plasma viral loads into the chronic phase of infection and consequential pathogenesis leading to AIDS-defining clinical endpoints, CD8 depletion is not required for initial viral infection or high levels of acute viral replication.^22^^,^^23^ These properties make the stHIV-A19/PTM model suitable for evaluation of interventions intended to prevent initial viral infection. Importantly, the genome of stHIV-A19 is composed of 94% HIV-1 sequence, including an intact HIV-1 *gag* gene encoding an HIV-1 CA protein.

Here, we first defined both the in vitro susceptibility of stHIV-A19 replication to LEN inhibition and the PK of a long-acting formulation of LEN in PTMs. We then evaluated LEN PrEP in PTMs against a stringent high-dose stHIV-A19 challenge via the intravenous (IV) route, a transmission route estimated to account for approximately 10% of new HIV-1 infections.^24^ Finally, in light of recent reports of emergent HIV-1 infection characterized by drug-resistant virus in the setting of waning drug levels in a rare subset of individuals that received CAB-LA PrEP,^25^ we utilized experimental depletion of CD8+ cells, performed after LEN washout, to definitively confirm prevention of infection in animals that received LEN PrEP.

## Methods

### Drugs and formulation

Lenacapavir and a generic internal small-molecule standard used for the LEN bioanalysis experiments were both synthesized at Gilead Sciences (Foster City, CA) and subjected to a standard quality control analysis. For animal dosing studies, LEN was formulated as a sterile, preservative-free, injectable solution (309 mg/ml in 57.5% w/v polyethylene glycol 300 in water) and stored at ambient temperature, protected from light, until dosing.

### Viruses and antiviral assays

Infectious stocks of stHIV-A19,^23^ HIV-1 NL4-3, and SIVmac239 were produced in HEK-293T (RRID: CVCL_1926) cells transfected with full-length viral genome plasmids using the TransIT HEK-293 transfection reagent (Mirus Bio) according to the manufacturer’s instructions. Virus stock infectious titres were determined on TZM-bl reporter cells (RRID: CVCL_B478) as previously described.^26^ For experiments using SupT1-CCR5 target cells (RRID: CVCL_X633),^27^ cultures were maintained in RPMI 1640 medium supplemented with 10% foetal bovine serum (FBS), 2 mM glutamine, 100 U/ml penicillin, and 100 μg/ml streptomycin. SupT1-CCR5 cells were inoculated with stHIV-A19, HIV-1 NL4-3, or SIVmac239 at a multiplicity of infection (MOI) = 0.0125 and cultured at 37 °C for 4 h. Cells were then washed three times to remove input virus, counted, and cultured in triplicate in growth media containing serial threefold dilutions of LEN or no LEN (control). The concentration of DMSO (LEN vehicle) was maintained at 0.5% for all cultures. Culture supernatants were collected 7 days post-inoculation, clarified by centrifugation, and stored at −80 °C for later quantification of vRNA by quantitative reverse transcription polymerase chain reaction (qRT-PCR). Primary human peripheral blood mononuclear cells (PBMCs) from anonymous HIV-negative donors were isolated from leukopaks obtained from the New York Blood Center for experiments using primary human and PTM PBMC. Human and PTM PBMC cultures were maintained in RPMI 1640 medium supplemented with 10% FBS, 50 μg/ml gentamicin, and 20 U/ml interleukin 2 (IL-2) (Peprotech). Viably cryopreserved PBMCs were quickly thawed at 37 °C, washed with RPMI, and activated by resuspension in growth media containing 5 μg/ml phytohemagglutinin (PHA-P, Sigma-Aldrich). After 48 h at 37 °C, the PBMCs were transferred to fresh culture media containing infectious stHIV-A19 and serial threefold dilutions of LEN. The concentration of DMSO (LEN vehicle) was maintained at 0.2% for all cultures. The stHIV-A19 stock was quantified with a SYBR Green-based qPCR-based product-enhanced RT (PERT) assay using a purified reverse transcriptase (RT) protein standard, as previously described.^28^ For LEN inhibition experiments, a virus volume equivalent to 5 pg RT was added per 10^5^ cells. One day post inoculation, the PBMCs were washed three times with RPMI to remove input virus and resuspended in fresh growth media containing LEN at the same concentration as at the time of virus inoculation. After 7 days, the virus in culture supernatants was quantified using the PERT assay. The dose-response experiment was performed in independent cultures using cells from 3 separate donors, with each condition performed in triplicate for each donor. The mean 50% effective concentration (EC_50_) value for LEN vs. each virus was calculated from the dose-response curves generated using data from three independent experiments using a 4-parameter curve fit in GraphPad Prism 9.4.1 software. The EC_95_ for LEN against stHIV-A19 was then calculated from its corresponding EC_50_ values using the EC_anything_ equation: EC_F_ = EC_50_ × (F/100-F)^1/*n*^, where F equals the percent response (e.g., 95) and *n* equals the Hill coefficient for LEN (*n* = 3.51) as previously described.^16^

### Animals and treatments

**Supplementary Figure S1:**
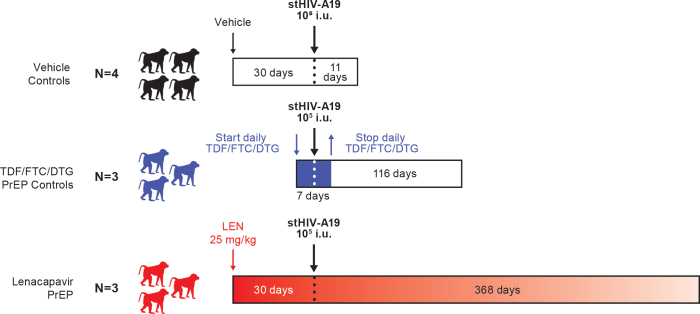
**Intravenous challenge study schema.** Study design, including relative timing of vehicle injection, lenacapavir (LEN) injection, initiation (blue downward arrow) and discontinuation (blue upward arrow) of daily oral TDF/FTC/DTG administration, and intravenous stHIV-A19 challenge. Following stHIV-A19 challenge, each group was monitored for the indicated duration. Animals in the vehicle control and TDF/FTC/DTG control groups were followed for a shorter duration than the LEN groups following stHIV-A19 challenge because they were repurposed for other studies to conserve animal resources. I.U., infectious units.

Sixteen male, purpose-bred pigtail macaques (*Macaca nemestrina*; ages 4.6–8.5 years at the time of study initiation) were housed at NIH-Bethesda (Supplementary Table S1). All study animals received enrofloxacin (10 mg/kg, administered orally, once per day for 10 days), paromomycin (25 mg/kg, administered orally, twice daily for 7–10 days), and fenbendazole (50 mg/kg administered orally, once per day for 5 days) two weeks to 43 months prior to study initiation. Lenacapavir or drug vehicle (sterile, preservative-free 57.5% w/v polyethylene glycol 300 in water) doses were administered to sedated animals via SC injection into the scapular region such that no more than 2 ml of solution was injected into a single SC site. Animals in the LEN pharmacokinetic evaluation study (N = 6) each received two doses of LEN, both at 15 mg/kg (N = 3) or at 50 mg/kg (N = 3), 6 weeks apart. Animals in the virus challenge experiment each received a single dose of LEN at 25 mg/kg (N = 3, LEN PrEP group) or drug vehicle (N = 4, vehicle control group) 30 days prior to stHIV-A19 challenge, or a cART regimen consisting of tenofovir disoproxil fumarate (TDF), emtricitabine (FTC), and dolutegravir (DTG) (N = 3, TDF/FTC/DTG group), coformulated and administered as previously described^29^^,^^30^ for 7 days beginning 3 days prior to stHIV-A19 challenge (Supplementary Figure S1). Hair was clipped and skin marked at LEN and vehicle injection sites to allow for subsequent visual and manual assessment and scoring of potential ISRs by non-blinded veterinary care staff. Complete blood counts (CBC) and serum chemistries were monitored prior to and at 2- to 4-week intervals after LEN administration for at least one year. Following the first dose of LEN in the PK study, one animal in the 50-mg/kg dosing group received 7 consecutive days of daily ketoprofen (2 mg/kg) administered intramuscularly for ISR analgesia at veterinary discretion. For the second dose of LEN in the PK study, all animals in the 50-mg/kg dosing group received 5–7 consecutive days of twice daily oral carprofen (2 mg/kg) analgesia administered prophylactically starting on the day of LEN dosing. In the IV challenge PrEP study, all LEN and vehicle control animals received 3–5 consecutive days of twice daily oral carprofen (2 mg/kg) analgesia administered prophylactically starting on the day of injection. For virus challenge, PrEP study animals each received 1 × 10^5^ infectious units of stHIV-A19 injected intravenously. After LEN washout, LEN PrEP group animals received a single 50-mg/kg dose of rhesusized depleting anti-CD8α antibody (MT807R1, RRID: AB_2716320, NIH Nonhuman Primate Reagent Resource), administered SC to anesthetized animals.

### Specimen collection and sample preparation

Whole blood was collected in EDTA Vacutainer Tubes (BD) from sedated animals. Plasma for vRNA quantification, LEN concentration determination, and HIV-1 Western blot analysis was separated from whole blood and clarified by centrifugation, and then stored at −80 °C. Following plasma separation, the blood cellular component was resuspended in an equivalent volume of PBS and then PBMCs were isolated by Ficoll-Paque Plus (GE Healthcare) gradient centrifugation. Portions of isolated PBMC samples were cryopreserved as dry cell pellets for CA-vDNA quantification.

### Viral load quantification

Simian-tropic HIV was isolated from clarified PTM plasma by centrifugation (21,000×*g* for 1 h), and the resultant pellet was extracted as described.^31^ Pigtail macaque PBMCs were subjected to nucleic acid extraction essentially as previously described for tissues.^32^ HIV *gag* DNA and RNA copies were assessed using a multiplexed qPCR assay, as previously described.^33^ Plasma stHIV-A19 vRNA levels are reported as copies/ml of plasma. PBMC-associated stHIV-A19 vDNA levels are reported as copies/10^6^ cell equivalents, based on 2 copies of the CCR5 gene per diploid cellular genome.^34^

### HIV-1 Western blots

Plasma specimens collected from all 10 virus challenge animals prior to and at 8 weeks after stHIV-A19 challenge were evaluated for the presence of antibodies specific for HIV-1 by Western blot analysis using the GS HIV-1 Western Blot kit (Bio-Rad, cat #32508) according to the manufacturer’s instructions.

### Bioanalysis of LEN in PTM plasma

Pigtail macaque plasma samples stored frozen at −80 °C were thawed, and a 50 μl aliquot of each was treated with 200 μl acetonitrile containing a generic internal small-molecule standard. After precipitation of the protein component, a 150 μl aliquot of the supernatant was transferred to a clean 96-well plate and evaporated to dryness under a nitrogen stream. The samples were reconstituted with 100 μL of 50% acetonitrile and then injected into an API-6500+ QTRAP triple quadrupole mass spectrometer (SciEx) with electrospray ionization in positive mode. Quantification was performed using multiple reaction monitoring of the transitions *m/z* 968.1–868.5 and *m/z* 756.3–600.2 for LEN and the generic internal standard, respectively. The lower and upper limits of quantitation for LEN were 0.1 nM and 1000 nM, respectively. Assay accuracy ranged from 81.9% to 109.0%. The descriptive pharmacokinetic parameters were analysed using noncompartmental analysis in Phoenix WinNonlin software (version 8.2, Certara USA, Inc.). The peak concentration (C_max_) and time to reach peak concentration (T_max_) values were recorded directly from experimental observations. The area under the plasma concentration–time curve (AUC_last_) values were calculated using the standard trapezoid method. The area under the plasma concentration-time curve (AUC_inf_) values were calculated as AUC_last_ + C_last_/elimination rate (kel). Terminal half-life (t_1/2_) values were calculated as natural log (2)/kel. Kel values were calculated using linear regression analysis of selected time points in the apparent terminal phase of the log plasma concentration vs. time curve (not reported).

### Equilibrium dialysis shift assay

The protein shift ratio for LEN in PTM plasma was determined by competitive equilibrium dialysis. Lenacapavir (2 μM) was spiked into 10% PTM plasma from each of 4 individual donors (3 males and 1 female) and then placed in triplicate into one side of assembled dialysis cells (Harvard Apparatus), followed by the addition of RPMI cell culture medium containing 10% foetal bovine serum to the opposite side of each cell. After a 24-h equilibration period at 37 °C, plasma and cell culture medium samples were collected and individually mixed 1:1 with the reciprocal blank matrix. Samples were then deproteinated following the addition of acetonitrile (1:4 ratio, respectively) containing 50 ng/ml warfarin and 50 ng/ml ritonavir as an internal standard and analysed using a liquid chromatography with tandem mass spectrometry (LC-MS/MS) method utilizing a SciEx API 4000 triple quadrupole mass spectrometer equipped with a Shimadzu Nexera UPLC system. The mean plasma protein binding-adjusted shift for LEN in 100% PTM plasma obtained from 4 independent experiments, each using a single PTM plasma donor performed in triplicate, was then calculated using a non-linear equation.

### Flow cytometric analysis for CD8 depletion

Absolute cell counts of immune cell populations in whole blood were determined by flow cytometry. Antibodies and reagents were obtained from BioLegend, unless indicated otherwise, and data analysis was performed using FCS Express (De Novo Software). Antibody panel validation and population gating were performed using fluorescence-minus-one and corresponding biological controls, instrument calibration was performed daily with NovoCyte 6 peak QC particles and compensation was performed for each study timepoint using goat-anti-mouse Ig (Spherotech) compensation bead, single-color controls, under identical sample treatment conditions. For the assay, 50 μL EDTA–anti-coagulated whole blood was incubated for 20 min with 50 μL of the following titrated and optimized antibodies: CD20 Pacific Blue (clone 2H7; RRID: AB_493651), CD28 Brilliant Violet (BV) 510 (clone CD28.2; RRID: AB_2562030), CD45 Brilliant Ultra Violet (BUV) 805 (clone DO58-1283, BD; RRID: AB_2871344), CD16 BV785 (clone 3G8; RRID: AB_2563803), CD123 PE-Dazzle 594 (clone 6H6; RRID: AB_2566450), CD11c APC (clone S-HCL-3; RRID: AB_2616902), CD137 BV650 (clone 4B4-1; RRID: AB_2572193), CD56 BUV615 (clone B159, BD; RRID: AB_2875357), HLA-DR FITC (clone L243; RRID: AB_1089142), CD8b BUV496 (clone 2ST8.5H7, BD; AB_2874080), CD3 PE (clone SP34-2, BD; AB_394342), CD95 PE-Cy5 (clone DX2; RRID: AB_314548), CD8α PE-Cy7 (clone SK1; RRID: AB_2044008), CD4 BV711 (clone OKT4; RRID: AB_2562912), and CD14 APC-Fire 750 (clone M5E2; RRID: AB_493695). Samples were then incubated for 10 min with 2 ml of 1X 1-Step Fix/Lyse Solution (eBioscience), and then approximately ½ of the total volume was acquired for each sample on a NovoCyte Penteon (Agilent) flow cytometer equipped with a calibrated, high-precision syringe pump, for accurate measurement of sample acquisition volume. Cell counts per microliter of whole blood were calculated using the following formula: [# cells/μL whole blood = (# gated cells/50 μL input whole blood∗2100 μL total sample volume)/sample volume acquired (μL)].

### Animal care

Animals enrolled on the LEN PK study were socially housed with other animals, when compatible, with oversight from facility behavioural management staff. Study AVP-078 had an ACUC-approved exemption from social housing for animals involved in the stHIV-A19 challenge experiments based on scientific justification and were housed in individual primate cages allowing interactions (visual and auditory) but no direct contact. Primary enclosures consisted of stainless-steel primate caging provided by a commercial vendor. Animal body weights and housing in cages of appropriate dimensions were regularly monitored. Abnormalities noted during study procedures or during the regular care of the animals was brought to the attention of the veterinary staff. Overall dimensions of primary enclosures (floor area and height) met or exceeded the specifications of The Guide for the Care and Use of Laboratory Animals, and the Animal Welfare Regulations (AWRs). Further, all primary enclosures were sanitized every 14 days at a minimum, in compliance with AWRs. Primary enclosures were contained within animal rooms under light (12-h light/12-h dark light cycle), temperature, humidity, and airflow monitoring using building automated controls. Animals were fed commercial monkey chow, twice daily, with supplemental enrichment food items provided daily, including, but not limited to, fruit or other produce at least three times per week. Filtered, chlorinated water was available ad libitum. Animals were observed at least twice daily by trained personnel, including behavioural assessments. Environmental enrichment included provision of species appropriate manipulatives, and foraging opportunities, as well as auditory (music) and visual (video watching) enrichment multiple times per week. The ACUC-approved humane endpoint criteria for the study included: 1) weight loss >15% body weight in 2 weeks or 20% body weight in 2 months or 25% overall, 2) documented opportunistic infection, 3) persistent anorexia >3–5 days without explicable cause, 4) severe intractable diarrhoea that was nonresponsive to standard treatment and resulted in dehydration and debilitation of the animal, 5) progressive neurologic signs (i.e., instability on the perch bar, head tilt, nystagmus, ataxia, stupor, or depression), 6) significant cardiac and/or pulmonary signs (i.e., dyspnoea, open-mouthed breathing, severe, previously unrecognized, cardiac murmur especially if resulting in pulmonary oedema), 7) persistent leukopenia (as a general guideline defined as <1000 cells/L) or thrombocytopenia (as a general guideline defined as <30,000 platelets/L), 8) progressive or persistent anaemia (<20% haematocrit), 9) CD4 depletion or other signs of progressive immunosuppressive disease, 10) body condition score <1.5/5 with weight loss, 11) any other serious illness. In addition to these specific criteria, the decision to euthanize any animal rested with the professional judgment of the veterinary staff, including due to the culmination of signs not directly related to those enumerated above.

### Sample size

Sixteen total pigtail macaques were used for these studies. In addition to the considerations listed below, study design was based in part on the practical feasibility of animal housing and care, and specimen processing and analysis requirements. Six animals were used for the LEN pharmacokinetic evaluation study (N = 3 at each of two dose levels evaluated). Ten animals, separated into 3 study groups were used in the IV challenge study. The IV challenge study was designed to detect a reduction in risk of stHIV-A19 acquisition of approximately 70–90% while utilizing the minimum number of animals. A power analysis assessing the detectable differences in infection rate for the vehicle control group (N = 4) compared with the LEN PrEP (N = 3) or TDF/FTC/DTG control PrEP (N = 3) groups was performed. With a negative control group sample size of 4 and assuming a negative control infection rate of 99.5% or higher, 3 treated animals provide 80% power to detect an 82.2% or greater reduction in treated animal infectivity with alpha (p-value cutoff for significance) set at 0.05. Power calculations were performed using the “pwr.2p2n.test” function from the “pwr” package (Stephane Champely (2020). pwr: Basic Functions for Power Analysis. R package version 1.3-0, https://CRAN.R-project.org/package=pwr).

### Inclusion and exclusion criteria

Inclusion criteria for pigtail macaques enrolled onto the study were: male sex, age greater than 4 years and less than 10 years, and prior negative serologic testing for cercopithecine herpesvirus 1, SIV, simian type-D retrovirus, and simian T lymphotropic virus type 1.

### Randomisation

Animals were not randomly assigned; they were assigned to study groups based on veterinary behavioural assessments for acceptance of conscious daily subcutaneous drug administrations and animal housing requirements. Batched molecular virologic analyses of specimens from all treatment groups were performed to obviate potential confounding from order of measurements. In the IV challenge study, all LEN and vehicle control animals received oral carprofen analgesia to avoid potential confounding effects of imbalanced analgesia use. Other potential study confounders were not controlled for.

### Blinding

All researchers involved with the animal study, data generation, and data analysis were aware of animal group assignments.

### Ethics

All work involving research animals was conducted under a protocol (AVP-078) approved by the Animal Care and Use Committee of the National Cancer Institute, National Institutes of Health (NIH) in NIH-Bethesda facilities. NIH-Bethesda is accredited by AAALAC International and follows the Public Health Service Policy for the Care and Use of Laboratory Animals (Animal Welfare Assurance Number D16-00602). Animal care adhered to the standards outlined in the “Guide for the Care and Use of Laboratory Animals (National Research Council; 2011; National Academies Press; Washington, D.C.), in accordance with the Animal Welfare Act.

### Statistics

Statistical analyses were performed using R v4.1.1 ((R Core Team, 2021). R: A language and environment for statistical computing. R Foundation for Statistical Computing, Vienna, Austria. URL https://www.R-project.org/). Infection rates between the vehicle control group and the LEN PrEP group or the TDF/FTC/DTG control group were compared using Fisher’s exact test performed using the fisher.test function from the base “stats” package of R, with a two-sided p-value <0.05 considered significant.

### Role of funders

Scientific staff at Gilead Sciences, Inc., participated in study design, pharmacokinetics data generation, data analysis, data interpretation, and writing of the report. Gilead Sciences, Inc., reviewed the draft of the manuscript. Other funders did not participate in study design, data generation, data analysis, data interpretation, or manuscript writing.

## Results

### Model assessment

**Fig. 1:**
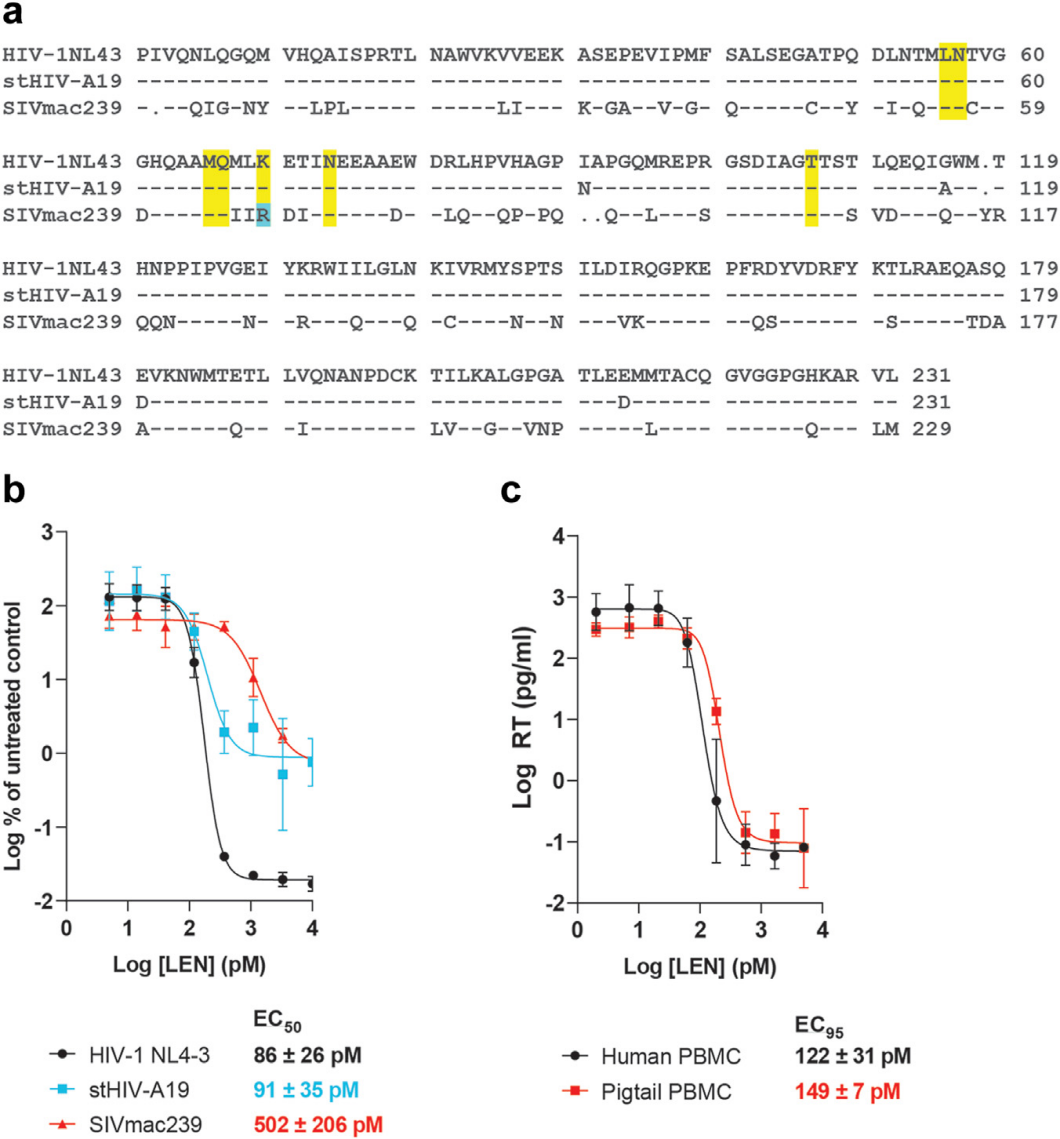
**Sensitivity of stHIV-A19 to LEN. (a)** Alignment of capsid protein (CA) amino acid sequences for HIV-1 NL4-3, stHIV-A19, and SIVmac239. Locations of known amino acid substitutions conferring LEN resistance are highlighted in yellow. An amino acid difference in the SIVmac239 CA sequence associated with reduced LEN sensitivity for HIV-1 is highlighted in blue. **(b)** In vitro antiviral dose response curves for LEN against HIV-1 NL4-3, stHIV-A19, or SIVmac239 in multi-round viral replication assays in SupT1-CCR5 cells. Viral RNA content in culture supernatants collected at 7 days post-infection was quantified and expressed as a percentage of vRNA content in no-drug control cultures. Lenacapavir mean EC_50_ values ± SD determined against each virus are shown. **(c)** In vitro antiviral dose response curves for LEN against stHIV-A19 in multi-round viral replication assays in primary human and pigtail macaque PBMCs from three independent donors. Viral reverse transcriptase (RT) protein was quantified in culture supernatants collected 7 days post-infection. Calculated EC_95_ values for LEN against stHIV-A19 in each cell type are shown. Data in **(b)** and **(c)** represent means ± SD values from three independent experiments each performed in triplicate.

To assess the relevance of the stHIV-A19 model compared with other NHP lentiviral infection models for evaluation of LEN PrEP, we first compared the amino acid sequence of the CA protein of the prototypical HIV-1 NL4-3 infectious molecular clone with stHIV-A19 and the SIVmac239 infectious molecular clone. As shown in Fig. 1a, SIVmac239 CA possessed two amino acid deletions, one amino acid insertion, and 74 amino acid differences, including one associated with LEN drug resistance (K70R)^16^ when compared with the HIV-1 NL4-3 CA (67% sequence identity). Conversely, stHIV-A19 and HIV-1 NL4-3 CA sequences differed at only 4 positions (98% sequence identity) with no substitutions that have previously been associated with LEN drug resistance.

We compared LEN potency against HIV-1 NL4-3, stHIV-A19, and SIVmac239 replication in a multi-round in vitro infection assay in SupT1-CCR5 cells,^27^ a human T cell line that supports replication of all three viruses. Because it is an extensively lab-adapted viral clone, NL4-3 replicated to ∼100-fold higher levels than either stHIV-A19 or SIVmac239 in SupT1-CCR5 cells, resulting in greater available dynamic range for the maximal magnitude of inhibition of NL4-3. Consistent with the high degree of sequence identity between their CA proteins, stHIV-A19 and HIV-1 NL4-3 were comparably sensitive to LEN. In comparison, LEN showed approximately 6-fold reduced potency against SIVmac239, with a mean EC_50_ value of 502 (±206 SD) pM as compared to 86 (±26 SD) pM and 91 (±35 SD) pM against HIV-1 NL4-3 and stHIV-A19, respectively (Fig. 1b).

To provide interpretive context for PK analyses of LEN in PTMs, we determined the PA-EC_95_ for LEN inhibition of stHIV-A19 by first assessing LEN antiviral potency against stHIV-A19 in a multi-round replication assay conducted in both primary human and PTM PBMCs (Fig. 1c). Lenacapavir was similarly potent in both human and PTM PBMCs with mean EC_95_ values of 122 (±31 SD) and 149 (±7 SD) pM, respectively, indicating that key cellular host factors involved in the multimode mechanism of action of LEN^16^ are likely conserved between these two species. Based on competitive equilibrium dialysis studies, LEN in vivo antiviral potency was projected to be 9.7-fold reduced in the presence of 100% PTM plasma as compared to complete cell culture media, resulting in a PA-EC_95_ value of 1.46 (±0.07 SD) nM for LEN against stHIV-A19 in PTMs.

### Lenacapavir pharmacokinetics in PTMs

**Fig. 2:**
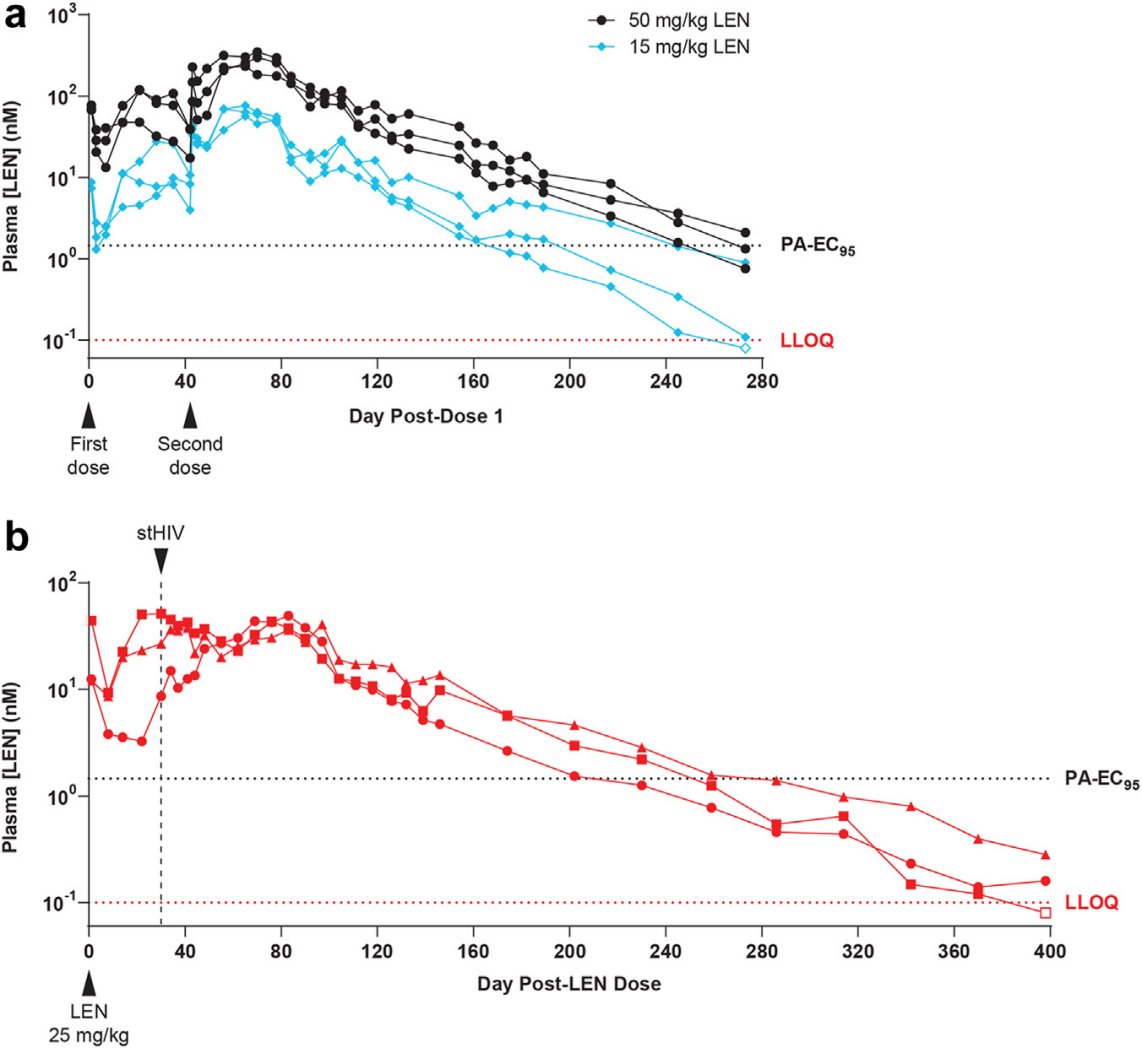
**Pharmacokinetics of subcutaneous LEN in pigtail macaques.** Longitudinal plasma LEN concentrations measured by liquid chromatography mass spectrometry (LC-MS) in **(a)** naïve pigtail macaques that received two doses of LEN at 15 mg/kg (N = 3; blue diamonds) or 50 mg/kg (N = 3; black circles) 6 weeks apart and in **(b)** three pigtail macaques in the LEN PrEP study group that each received a single subcutaneous injection of LEN at 25 mg/kg and were intravenously challenged with stHIV-A19 30 days later. In each plot, the bottom horizontal red dashed line represents the assay lower limit of quantification (LLOQ; 0.1 nM) and the top horizontal black dashed line represents the plasma protein binding-adjusted EC_95_ (PA-EC_95_; 1.46 nM) for LEN against stHIV-A19 in pigtail macaque peripheral blood mononuclear cells.

To investigate the PK of a long-acting formulation of LEN in PTMs, six naïve PTMs each received two weight-adjusted SC LEN injections six weeks apart. Both LEN doses were administered at either 15 mg/kg (N = 3) or 50 mg/kg (N = 3). These LEN doses were selected based on limited data obtained from a prior small LEN PK assessment in cynomolgus macaques (unpublished). As expected, LEN displayed long-acting PK with “flip-flop” kinetics (Fig. 2a) characteristic of sustained release drug formulations.^35^, ^36^, ^37^, ^38^ Following the first dose of LEN, plasma drug concentrations peaked 2–5 weeks post-dose with mean plasma LEN concentrations of 16 nM (range 10–28 nM) and 95 nM (range 48–120 nM) for the 15 mg/kg and 50 mg/kg groups, respectively (Fig. 2a). Having documented the magnitude and timing of peak LEN concentrations in plasma, which occurred within the first 5 weeks after a single dose of LEN, we administered a second dose of LEN to each animal to determine if a second dose could help maintain elevated drug levels for a longer period and to provide additional tolerability data following LEN re-administration in this NHP species. Following the second dose of LEN, plasma drug concentrations peaked at 2–4 weeks post-dose at higher levels than those measured following dose 1, with mean plasma LEN concentrations of 68 nM (range 57–77 nM) and 295 nM (range 234–350 nM) for the two groups, respectively (Fig. 2a and Table 1). Plasma drug levels declined slowly following the second dose of LEN, with a mean terminal half-life (t_1/2_) of 48 days and mean plasma drug concentrations above the PA-EC_95_ for >245 days in the 50 mg/kg group and a mean t_1/2_ of 42 days and mean plasma drug concentrations above the PA-EC_95_ for >189 days in the 15 mg/kg group.

**Table 1:** Pharmacokinetic parameters for pigtail macaques dosed with subcutaneous LEN.

Pharmacokinetic Parameter	15 mg/kg LEN^a^ (N = 3)		50 mg/kg LEN^a^ (N = 3)		25 mg/kg LEN^b^ (N = 3)	
	Mean ± SD	CV%	Mean ± SD	CV%	Mean ± SD	CV%
AUC_last_ (μM∗h)	80.7 ± 2.5	3.1	408 ± 106	26.1	91.3 ± 17.8	19.5
AUC_inf_ (μM∗h)	80.9 ± 2.5	3.1	408 ± 106	26.1	92.0 ± 17.8	19.3
t_1/2_ (h)	1010 ± 676	67.0	1160 ± 140	11.9	925 ± 342	36.9
C_max_ (nM)	67.8 ± 10.1	14.9	295 ± 58	19.8	47.0 ± 5.7	12.1
T_max_ (h)	1570 ± 84	5.4	1640 ± 69	4.2	1520 ± 898	59.1

Analytes include area under the plasma concentration-time curve from time 0 to the last quantifiable time point (AUC_last_), area under the plasma concentration-time curve from time 0 to infinity (AUC_inf_), maximal concentration (C_max_), time to reach observed peak plasma concentration (T_max_) and terminal half-life (t_1/2_), determined by non-compartmental analysis using Phoenix WinNonlin 6.4 build 8.1.03530.

CV, coefficient of variation (100 × standard deviation/mean).

a Sequential subcutaneous LEN dosing to pigtail macaques on Study Day 0 and 42.

b Single subcutaneous LEN dosing to pigtail macaques on Study Day 0.

No abnormalities or significant changes in complete blood counts (CBC) or blood serum chemistries were noted in animals that received LEN injections (data not shown). Mild to moderate injection site reactions (ISRs) were observed in some animals following some LEN injections or vehicle control injections. There were no clear associations between injection dose and development of ISRs in PTMs. Development of ISRs following dose 1 was not predictive of ISR development following dose 2 in the same animal (Supplementary Table S2).

### Protection from IV stHIV challenge with LEN PrEP

We next evaluated whether LEN PrEP could protect PTMs from a single IV challenge with 1 × 10^5^ infectious units (i.u.) of stHIV-A19. Naïve PTMs were treated with either LEN PrEP (N = 3), a vehicle control injection (N = 4), or a control PrEP regimen (N = 3) consisting of daily treatment with a three-drug combination antiretroviral therapy (cART) regimen (emtricitabine, tenofovir disoproxil fumarate, dolutegravir; TDF/FTC/DTG) (Supplementary Figure S1). For a control PrEP regimen, rather than utilize a clinically-employed PrEP regimen that has not previously been evaluated in the stHIV-A19/PTM model, we utilized a regimen that could be expected to effectively block stHIV-A19 infection in PTMs in vivo, allowing for unambiguous interpretation of any potential breakthrough infections that should occur in the LEN PrEP group. Accordingly, we chose a daily TDF/FTC/DTG regimen that we have previously administered safely to PTMs to effectively suppress stHIV-A19 replication in vivo (unpublished).

Based on the LEN dose/exposure relationship established during the first six weeks of the PK study, an intermediate LEN dose was selected to ensure sufficient plasma drug exposure at the time of high-dose IV challenge while also reducing the washout period relative to what would be necessary for a higher single dose of LEN to enable proper evaluation of LEN’s protective efficacy in an experimentally feasible time frame. The LEN PrEP animals each received a single 25-mg/kg LEN SC injection 30 days prior to stHIV-A19 challenge. Animals were challenged 30 days after LEN administration because this time point was projected to approximate maximal plasma LEN concentrations. At the time of stHIV-A19 challenge, plasma LEN concentrations were >5-fold above the PA-EC_95_ in all 3 animals, ranging from 8.6 to 51.2 nM (Fig. 2b). Drug levels declined slowly in these animals, with a mean t_1/2_ of 38.5 days and plasma LEN concentrations remaining above the PA-EC_95_ for >200 days and remaining above the lower limit of quantitation (LLOQ, 0.1 nM) for more than one year in all three animals following a single SC LEN administration (Fig. 2b and Table 1).

**Fig. 3:**
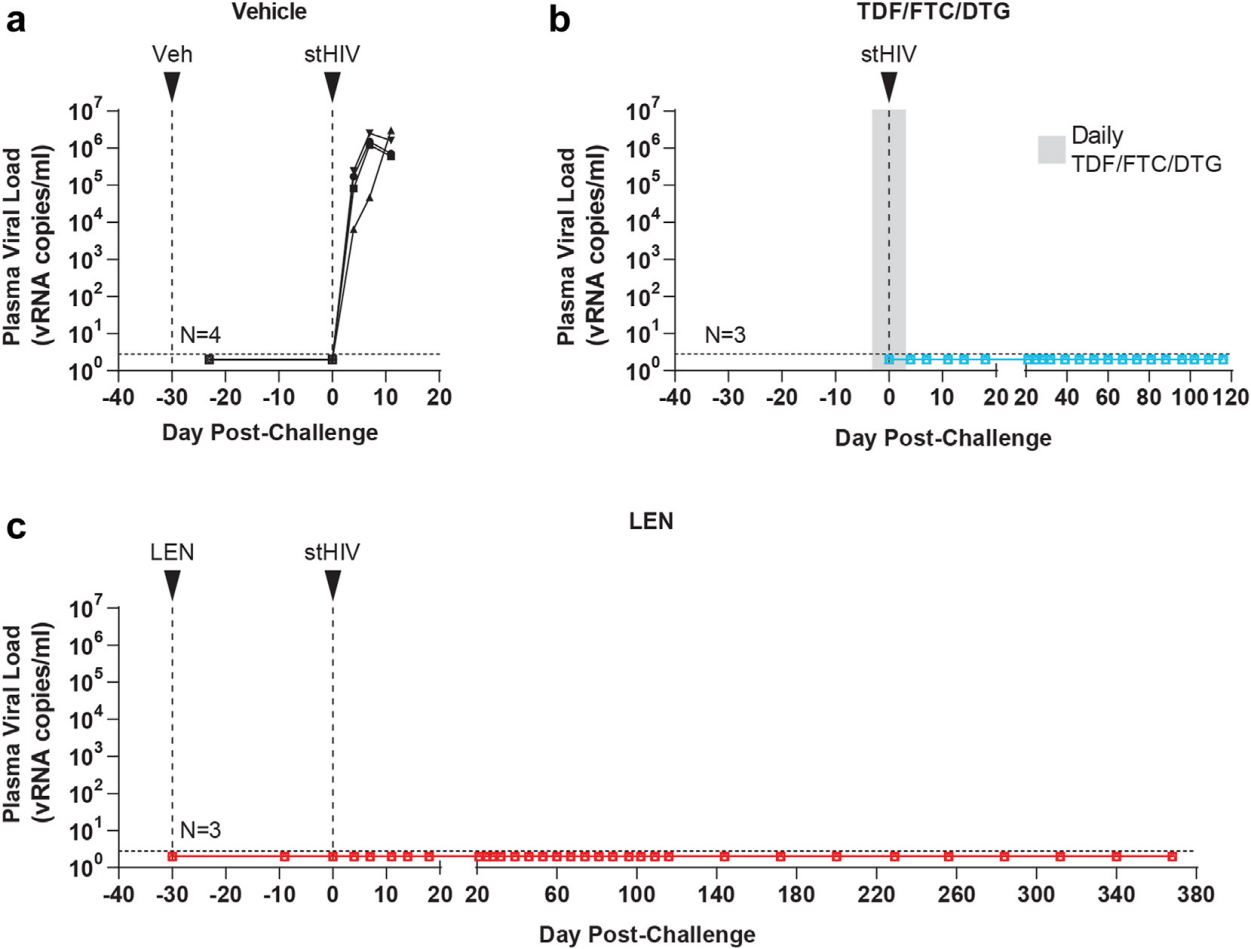
**Plasma viral loads following stHIV-A19 challenge.** Longitudinal plasma viral loads for **(a)** vehicle control, **(b)** TDF/FTC/DTG control PrEP, and **(c)** LEN PrEP group animals prior to and following intravenous stHIV-A19 challenge. The timing of vehicle, LEN, and stHIV-A19 injections are indicated by arrows. The grey shaded region in **(b)** indicates a 7-day period of daily TDF/FTC/DTG administration, beginning three days prior to stHIV-A19 challenge. Horizontal dashed lines indicate the assay quantification limit (2.8 vRNA copies/ml plasma). Plot symbols below the quantification limit represent specimens in which no viral RNA was detected.

We used an ultrasensitive plasma HIV-1 viral load assay with a threshold sensitivity of 2.8 viral RNA (vRNA) copies/ml to monitor plasma viral loads after stHIV-A19 challenge. All four vehicle control group animals were productively infected following stHIV-A19 challenge, with quantifiable plasma viral loads detected by 4 days post-challenge and increasing until peak viremia, ranging from 6 × 10^5^ to 3 × 10^6^ vRNA copies/ml at 11 days post-challenge (Fig. 3a). To conserve animal resources, the vehicle control animals were repurposed for another study following documentation of peak plasma viremia at day 11 post-challenge, definitively confirming take of infection. For the TDF/FTC/DTG PrEP control group, daily cART was administered via SC injection for seven days starting three days prior to stHIV-A19 challenge through three days post-challenge (Fig. 3b). In contrast to the vehicle control animals, plasma vRNA was not detected in the TDF/FTC/DTG PrEP control group at any time point through 120 days post-challenge (Fig. 3b). Plasma viral load results for the LEN PrEP group paralleled those of the TDF/FTC/DTG control group, with no plasma vRNA detected at any time point following stHIV-A19 challenge during ∼370 days of follow-up spanning the LEN washout period (Fig. 3c). Infection rates in the TDF/FTC/DTG PrEP control group and in the LEN PrEP group were each significantly lower than in the vehicle control group (each p < 0.03, Fisher’s exact test).

**Fig. 4:**
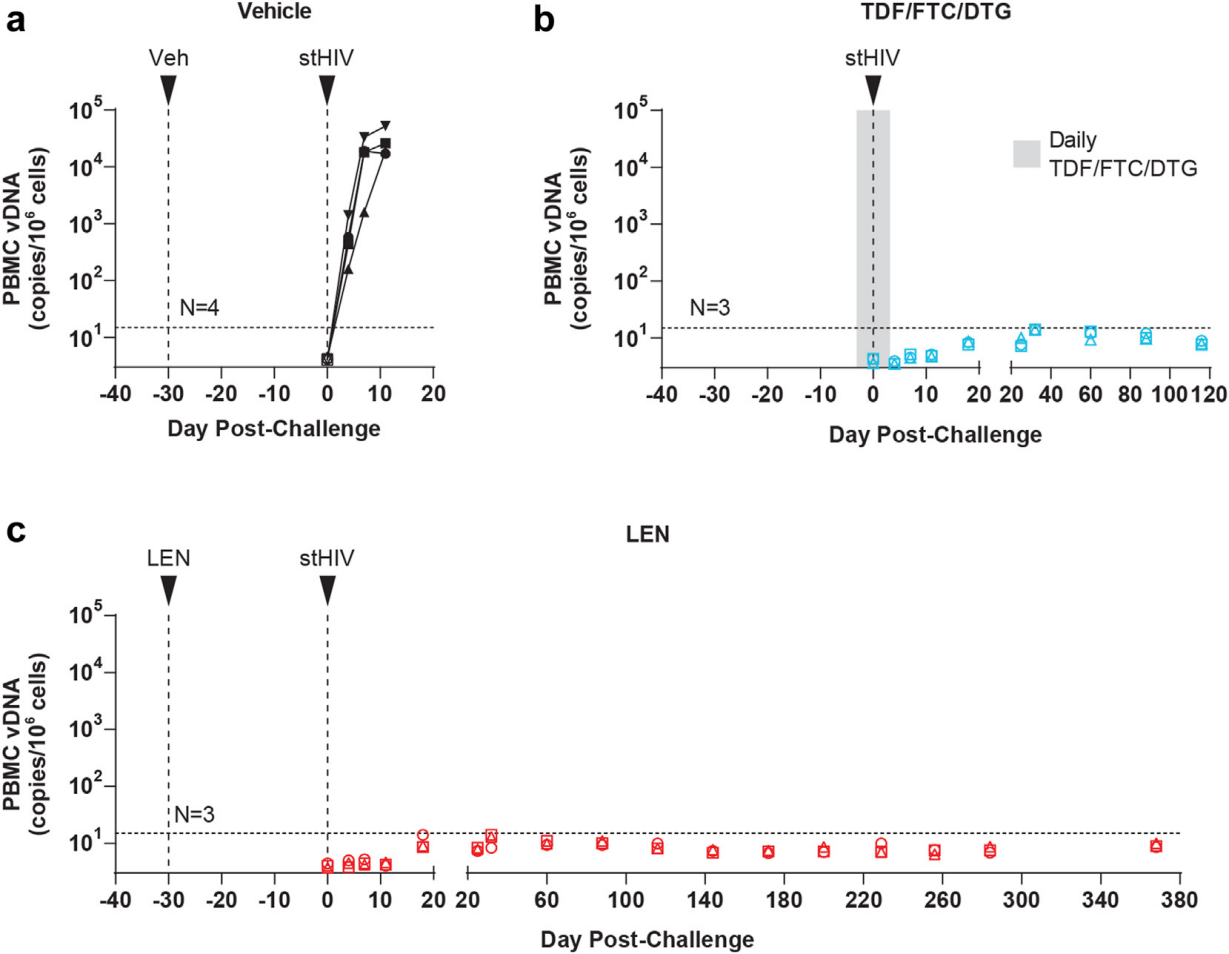
**Cell-associated viral DNA following stHIV-A19 challenge.** Cell-associated viral DNA (CA-vDNA) levels in PBMC for **(a)** vehicle control, **(b)** TDF/FTC/DTG control PrEP, and **(c)** LEN PrEP group animals prior to and following intravenous stHIV-A19 challenge. The timing of vehicle, LEN, and stHIV-A19 injections are indicated by arrows. The grey shaded region in **(b)** indicates a 7-day period of daily TDF/FTC/DTG administration, beginning three days prior to stHIV-A19 challenge. Determined vDNA levels were normalized based on input diploid genome cell equivalents assayed for each sample determined by the duplex quantification of the copy number of a host cell gene within the same sample extraction. Symbols are plotted at the threshold sensitivity limit for that sample based on the number of cell equivalents assayed. Horizontal dashed lines indicate the highest threshold assay quantification limit (based on the lowest cell input for the assay) across all analysed samples (15 vDNA copies/10^6^ cell equivalents). Plot symbols below the quantification limit represent specimens in which no vDNA were detected.

**Supplementary Figure S2:**
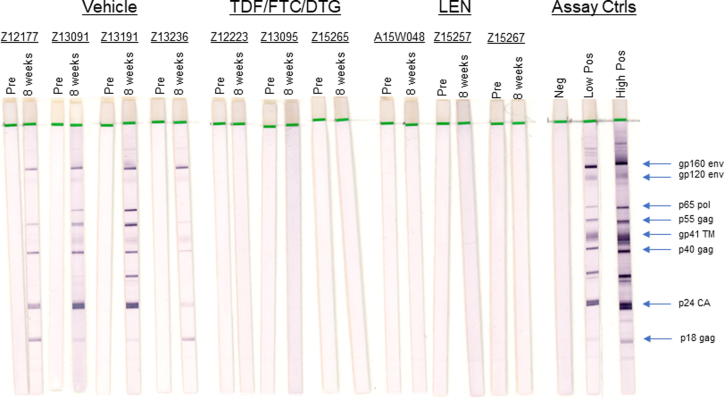
**Challenge study animal seroreactivity with HIV-1 Proteins.** Plasma samples collected prior to (“Pre”) or 8 weeks after intravenous stHIV-A19 challenge for all 10 challenge study animals were evaluated for the presence of antibodies specific for HIV-1 proteins by Western blot analysis.

We also longitudinally quantified cell-associated viral DNA (CA-vDNA) in PBMC collected prior to and following stHIV-A19 challenge in all three study groups (Fig. 4). Consistent with the plasma viral load results, in the vehicle control group animals, quantifiable PBMC vDNA was detected by 4 days post-challenge with levels increasing to >10^4^ vDNA copies/10^6^ cells through 11 days in all four animals (Fig. 4a). Conversely, vDNA was not detected at any time point in the TDF/FTC/DTG PrEP control animals (Fig. 4b) or any of the LEN PrEP animals (Fig. 4c). Serological assessments by HIV Western blot were consistent with molecular virologic analyses. Prior to stHIV-A19 challenge, plasma specimens from all 10 animals were non-reactive with HIV-1 proteins. At 8 weeks after stHIV-A19 challenge, all vehicle control animals seroconverted with HIV-1 antibodies detected in plasma, while all TDF/FTC/DTF PrEP animals and all LEN PrEP animals remained seronegative (Supplementary Figure S2). These data confirmed that productive infection was established in the vehicle control group but not in either the LEN PrEP group or the TDF/FTC/DTG PrEP control group.

### Experimental CD8 depletion in LEN PrEP animals

**Fig. 5:**
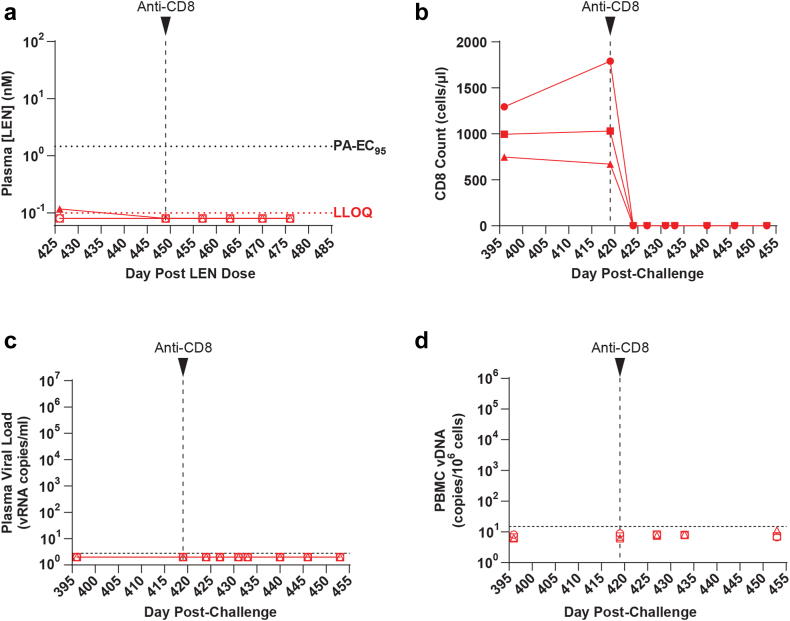
**Experimental depletion of CD8+ cells in LEN PrEP animals.** Animals in the LEN PrEP group received a single subcutaneous injection of the rhesusized anti-CD8 antibody MT807R1 (50 mg/kg) at the indicated time point, after LEN plasma concentrations declined below the lower limit of quantitation (LLOQ) in each of the three animals. **(a)** Longitudinal plasma LEN concentrations measured by LC-MS. The bottom horizontal red dashed line represents the assay LLOQ for LEN (0.1 nM). The top horizontal dashed black line represents the PA-EC_95_ (1.46 nM) for LEN against stHIV-A19 in pigtail macaque cells. **(b)** Longitudinal absolute CD8+ T cell counts in whole blood determined by flow cytometry. **(c)** Longitudinal plasma viral loads. The horizontal dashed line denotes the assay quantification limit (2.8 vRNA copies/ml plasma). Plot symbols below the quantification limit represent specimens in which no viral RNA was detected. **(d)** Longitudinal cell-associated viral DNA (CA-vDNA) levels in PBMC. Determined vDNA levels were normalized based on input diploid genome cell equivalents assayed for each sample determined by the duplex quantification of the copy number of a host cell gene within the same sample extraction. The horizontal dashed line indicates the maximum quantification limit across all analysed samples (15 vDNA copies/10^6^ cell equivalents). Plot symbols below the quantification limit represent specimens in which no vDNA was detected. Symbols are plotted at the threshold sensitivity limit for that individual sample based on the number of cell equivalents assayed.

Recent reports have noted rare breakthrough infections, characterized by the emergence of plasma virus containing drug resistance mutations, as drug levels waned in individuals that received CAB-LA PrEP.^25^^,^^39^ Although plasma vRNA was not detected in the LEN PrEP animals as LEN levels declined well below the PA-EC_95_, to definitively confirm that infection was prevented in the LEN PrEP animals, we administered an in vivo cell-depleting anti-CD8α antibody to the LEN PrEP animals at 419 days post-challenge, when LEN was no longer detected in plasma (Fig. 5a). We have previously shown that CD8 depletion with this antibody results in transient increases in plasma viral loads in stHIV-infected PTMs, including those with low levels of infection prior to depletion.^22^^,^^23^ Antibody-mediated CD8+ T cell depletion was robust in the LEN group animals, with CD8 T cell counts declining by 100% to 0 cells per μl blood in all 3 animals within 3 days of antibody administration (Fig. 5b). Following CD8 depletion, plasma vRNA and PBMC vDNA remained below the level of detection (<2.8 vRNA copies/ml plasma and <6.2–11.0 vDNA copies/10^6^ PBMC, respectively) in all 3 LEN PrEP animals during the subsequent 34 days of monitoring (Fig. 5c and d).

## Discussion

Pre-exposure prophylaxis can be highly effective for the prevention of HIV-1 infection. With optimal regimen adherence, daily oral PrEP is estimated to reduce the risk of sexual HIV-1 acquisition by ∼99%.^4^^,^^6^^,^^7^^,^^10^^,^^11^^,^^40^ However, PrEP effectiveness critically depends upon regimen adherence, and a wide range in rates of adherence have been reported for different populations and studies.^2^^,^^3^^,^^8^^,^^9^^,^^11^, ^12^, ^13^ The development of long-acting antiretroviral agents that require less frequent drug administration represents a promising approach to overcome some of the limitations associated with daily oral PrEP and would provide alternative options for the prevention of HIV-1 infection. In two large, randomized, double-blind, placebo-controlled clinical trials (HPTN 083, HPTN 084) comparing long-acting CAB-LA PrEP, administered intramuscularly once every 2 months, with daily oral FTC/TDF PrEP, there were significantly lower incidences of HIV-1 infection in the CAB-LA arms compared with the FTC/TDF arms, with the difference in efficacy likely attributable to non-adherence in some participants in the FTC/TDF study groups.^14^^,^^15^

Here, we show that a single SC administration of LEN, a first-in-class, long-acting inhibitor of HIV-1 CA function, protected PTMs from infection with a stringent, high-dose IV challenge of minimally chimeric HIV-1 (stHIV-A19). In all four vehicle control animals the same dose of stHIV-A19 resulted in productive infection, characterized by increasing plasma viral loads and increasing cell-associated viral DNA levels in PBMC, with both initially detected by 4 days post-challenge. We also show that LEN achieves prolonged plasma drug exposures in PTMS akin to those observed in humans that received LEN.^16^^,^^17^ At the time of stHIV-A19 challenge, performed 30 days after LEN administration, plasma LEN concentrations in the protected PTMs ranged from 5.9-fold to 35-fold above the LEN/stHIV-A19 PA-EC_95_. Of note, in a Phase 1b clinical trial, plasma LEN concentrations were maintained ≥6-fold above the PA-EC_95_ for 6 months in study participants that received a single SC injection of 900 mg LEN.^17^

Despite the clinical efficacy of CAB-LA PrEP, recent analyses of the HPTN 083 study have identified 7 breakthrough infections (among 2282 study participants in the CAB-LA arm) occurring in individuals that received on-time CAB-LA injections and had expected concentrations of CAB in plasma.^25^^,^^39^ These breakthrough infections initially went undetected by Ag/Ab testing methods, likely due to suppression of viral replication by CAB-LA resulting in reduced antigenemia and delayed seroconversion, which in turn may have allowed for selection and later emergence of virus containing CAB resistance mutations as CAB levels waned.^25^ Although in the current study neither vRNA nor vDNA emerged in blood in the LEN PrEP group following LEN washout, it remained possible that LEN initially suppressed rather than prevented infection, with subsequent immunologic control of viral replication by the time LEN washed out. To rule out this possibility, we utilized experimental CD8 depletion, an intervention that consistently induces viral load increases in stHIV infected animals, including in those exhibiting marked control of viral replication^22^ (unpublished results). Despite robust depletion of CD8+ T cells, stHIV-A19 vRNA in plasma and vDNA in PBMC remained below assay detection limits, definitively confirming that LEN prevented infection in the LEN group animals. Experimental CD8 depletion in macaques represents a powerful tool for accelerating the identification of potential low-level breakthrough infections should they occur in long-acting PrEP experiments conducted in NHPs.

We determined that LEN had an EC_50_ of 91 pM against stHIV-A19, which was virtually identical to the drug’s potency against HIV-1 NL4-3 in the same assay. While this finding is perhaps unsurprising given that the viral backbone of the stHIV precursor was derived from HIV-1 NL4-3,^22^^,^^41^ during its derivation via serial passage in pigtail macaques stHIV-A19 acquired four mutations in CA, which conferred partial resistance of the virus to the species-specific viral restriction factor Mx2.^23^ Our results here confirm that these mutations do not impact viral sensitivity to LEN. The potency of LEN against stHIV-A19 falls well within the range of LEN potency against primary clinical HIV-1 isolates from a range of viral subtypes, from 20 to 160 pM,^16^ indicating that stHIV-A19 is highly relevant for modelling LEN blockade of circulating HIV-1 variants.

A recent study evaluating GS-CA1, a structural analogue of LEN, found that a single administration of GS-CA1 protected rhesus macaques from repeated intrarectal challenges with a chimeric simian-human immunodeficiency virus (SHIV).^21^ In this model, GS-CA1 was shown to be 100% effective at preventing SHIV infection in animals challenged when plasma drug concentrations were more than twice the PA-EC_95_ for GS-CA1, with breakthrough infections only occurring when drug concentrations decayed below this level. Because SHIVs contain a SIV CA derived from SIVmac239, they are 5–8 fold less sensitive than HIV-1 to inhibition by capsid inhibitors such as GS-CA1 and LEN,^21^ and it is unclear if the relationship between plasma drug concentrations and protection from mucosal virus transmission tracks linearly with viral drug sensitivity. Follow up studies will involve utilization of an intrarectal stHIV-A19 transmission model in PTMs to assess LEN for PrEP against a mucosal stHIV-A19 challenge. Although the relationship between plasma drug concentrations and protection from sexual HIV-1 transmission will have to be defined by appropriately powered in-progress Phase 3 clinical trials (NCT04994509 and NCT04925752), the clinically relevant sensitivity of stHIV-A19 to LEN suggests that future studies examining protection from mucosal infection as a function of plasma drug concentrations in the stHIV-A19/PTM model may provide data to further explore and refine the drug exposure-efficacy relationship.

Limitations of this proof-of-concept study include the relatively small number of animals within each experimental group and the use of a single virus challenge performed when plasma LEN concentrations were at or near their highest levels. While this design enabled us to determine whether LEN could protect against a rigorous challenge using a relevant viral inoculum, it made it highly unlikely that we would detect any potential rare “breakthrough” infection events in PrEP group animals, such as those that have been documented in the clinic in individuals receiving CAB-LA PrEP. The study was powered to detect a reduction in risk of stHIV-A19 acquisition of at least 82.2% while minimizing the number of animals utilized. Notably, the US CDC estimates that for people who inject drugs (PWID) daily oral PrEP reduces the risk of HIV-1 acquisition by 74–84% with good regimen adherence.^8^^,^^9^^,^^40^ The use of an IV virus challenge may also represent a limitation of the study, as it is unclear whether or not tissue drug concentrations at the site of virus exposure, which may impact protection efficacy against mucosal virus challenge, similarly impact protection against IV virus challenge.

Although the majority of new HIV-1 infections occur via sexual transmission, IV transmission is estimated to account for approximately 10% of new HIV-1 infections.^24^ We therefore sought to assess the efficacy of LEN for PrEP against a high dose IV challenge in the stHIV-A19/PTM model as a particularly stringent proof-of-concept experiment. This inoculation route represents a notably rigorous challenge for approaches intended to block initial viral infection due to the relatively higher effective per-particle infectivity of challenge virus administered intravenously compared with mucosal challenge routes^42^ and because high-dose IV challenge, for which we have extensive experience in the stHIV-A19/PTM model, provides maximally broad, systemic viral exposure to potential sites of infection, including tissue sites of possible lower drug concentrations. While reduction of sexual HIV-1 transmissions will be critical for substantially reducing the overall incidence of HIV-1 infection, reduced transmission rates in PWID will also contribute meaningfully to efforts to reduce the public health impacts of the HIV pandemic. Our findings here support a planned Phase 2 clinical trial evaluating LEN for PrEP in PWID that are at increased risk of HIV-1 infection.

## Contributors

GQD, JDL, SRY, and WB contributed to the conceptualization of the study. GQD, JDL, SRY, WB, and AES contributed to the study design. GQD, AES, and JDL contributed to detailed study planning. GQD, JDL, SRY, and DD contributed to writing the primary manuscript draft, with input from all authors. GQD, RJG, BL, KW, FS, DD, PDB, TH, JAT, CMT, JZ, and SRY contributed to data analysis and interpretation. GQD, RJG, FS, JLW, BL, KW, MWB, KEK, JAK, JDR, TH, CP, JAT, CMT, and SRY contributed to the generation of the primary data. WR contributed to drug formulation. MWB, KEK, and JAK contributed veterinary care for all study animals. GQD, JDL, and SRY verified the underlying data generated by this study. Correspondence and requests should be addressed to SRY or GQD. The final version of this paper was reviewed and approved by all authors.

## Data sharing statement

Upon publication, the datasets presented here will be made available from the corresponding authors on reasonable request.

## Declaration of interests

BL, KW, WR, JZ, WB, and SRY are or were employees of Gilead Sciences, Inc., and received salary and stock ownership as compensation for their employment. WB, JZ, and SRY are inventors on patent application US20210188815A1 covering the use of capsid inhibitors for the prevention of HIV. JDL has served as a compensated advisor to Gilead Sciences, Inc., and JDL, GQD, and AES have received research support from Gilead Sciences, Inc., for studies unrelated to the work described here through separate collaborative research and development agreements with Leidos Biomedical Research, Inc. TH has received royalties from Wiley for textbook authorship, NIH honoraria for grant review panel participation, and registration fee reimbursement from Gordon conference for participating as an invited speaker. PDB receives salary support from the Howard Hughes Medical Institute and owns Gilead stock. The authors declare no other financial or competing interests exist.

## References

[1] UNAIDS (2021). http://wwwunaidsorg/en/resources/fact-sheet.

[2] Grant R.M., Lama J.R., Anderson P.L. (2010). Preexposure chemoprophylaxis for HIV prevention in men who have sex with men. N Engl J Med.

[3] Baeten J.M., Donnell D., Ndase P. (2012). Antiretroviral prophylaxis for HIV prevention in heterosexual men and women. N Engl J Med.

[4] Liu A.Y., Cohen S.E., Vittinghoff E. (2016). Preexposure prophylaxis for HIV infection integrated with municipal- and community-based sexual health services. JAMA Intern Med.

[5] Marcus J.L., Hurley L.B., Hare C.B. (2016). Preexposure prophylaxis for HIV prevention in a large integrated health care system: adherence, renal safety, and discontinuation. J Acquir Immune Defic Syndr.

[6] McCormack S., Dunn D.T., Desai M. (2016). Pre-exposure prophylaxis to prevent the acquisition of HIV-1 infection (PROUD): effectiveness results from the pilot phase of a pragmatic open-label randomised trial. Lancet.

[7] Marcus J.L., Hurley L.B., Nguyen D.P., Silverberg M.J., Volk J.E. (2017). Redefining human immunodeficiency virus (HIV) preexposure prophylaxis failures. Clin Infect Dis.

[8] Choopanya K., Martin M., Suntharasamai P. (2013). Antiretroviral prophylaxis for HIV infection in injecting drug users in Bangkok, Thailand (the Bangkok Tenofovir Study): a randomised, double-blind, placebo-controlled phase 3 trial. Lancet.

[9] Martin M., Vanichseni S., Suntharasamai P. (2015). The impact of adherence to preexposure prophylaxis on the risk of HIV infection among people who inject drugs. AIDS.

[10] Volk J.E., Marcus J.L., Phengrasamy T. (2015). No new HIV infections with increasing use of HIV preexposure prophylaxis in a clinical practice setting. Clin Infect Dis.

[11] Grant R.M., Anderson P.L., McMahan V. (2014). Uptake of pre-exposure prophylaxis, sexual practices, and HIV incidence in men and transgender women who have sex with men: a cohort study. Lancet Infect Dis.

[12] Marrazzo J.M., Ramjee G., Richardson B.A. (2015). Tenofovir-based preexposure prophylaxis for HIV infection among African women. N Engl J Med.

[13] Owens D.K., Davidson K.W., U. S. Preventive Services Task Force (2019). Preexposure prophylaxis for the prevention of HIV infection: US preventive services task force recommendation statement. JAMA.

[14] Landovitz R.J., Donnell D., Clement M.E. (2021). Cabotegravir for HIV prevention in cisgender men and transgender women. N Engl J Med.

[15] Delany-Moretlwe S., Hughes J.P., Bock P. (2022). Cabotegravir for the prevention of HIV-1 in women: results from HPTN 084, a phase 3, randomised clinical trial. Lancet.

[16] Link J.O., Rhee M.S., Tse W.C. (2020). Clinical targeting of HIV capsid protein with a long-acting small molecule. Nature.

[17] Begley R., Lutz J., Rhee M., Dvory-Sobol H., Chiu A., West S.K. (2020). Poster session presented at: AIDS 2020: the 23rd international AIDS conference; July 6-10, 2020; virtual..

[18] Segal-Maurer S., DeJesus E., Stellbrink H.J. (2022). Capsid inhibition with lenacapavir in multidrug-resistant HIV-1 infection. N Engl J Med.

[19] Margot N.A., Naik V., VanderVeen L. (2022). Resistance analyses in highly treatment-experienced people with human immunodeficiency virus (HIV) treated with the novel capsid HIV inhibitor lenacapavir. J Infect Dis.

[20] Yant S.R., Mulato A., Hansen D. (2019). A highly potent long-acting small-molecule HIV-1 capsid inhibitor with efficacy in a humanized mouse model. Nat Med.

[21] Vidal S.J., Bekerman E., Hansen D. (2022). Long-acting capsid inhibitor protects macaques from repeat SHIV challenges. Nature.

[22] Hatziioannou T., Del Prete G.Q., Keele B.F. (2014). HIV-1-induced AIDS in monkeys. Science.

[23] Schmidt F., Keele B.F., Del Prete G.Q. (2019). Derivation of simian tropic HIV-1 infectious clone reveals virus adaptation to a new host. Proc Natl Acad Sci U S A.

[24] UNAIDS (2020). https://www.unaids.org/en/resources/documents/2020/global-aids-report.

[25] Marzinke M.A., Grinsztejn B., Fogel J.M. (2021). Characterization of human immunodeficiency virus (HIV) infection in cisgender men and transgender women who have sex with men receiving injectable cabotegravir for HIV prevention: HPTN 083. J Infect Dis.

[26] Morcock D.R., Thomas J.A., Sowder R.C., Henderson L.E., Crise B.J., Gorelick R.J. (2008). HIV-1 inactivation by 4-vinylpyridine is enhanced by dissociating Zn(2+) from nucleocapsid protein. Virology.

[27] Means R.E., Matthews T., Hoxie J.A., Malim M.H., Kodama T., Desrosiers R.C. (2001). Ability of the V3 loop of simian immunodeficiency virus to serve as a target for antibody-mediated neutralization: correlation of neutralization sensitivity, growth in macrophages, and decreased dependence on CD4. J Virol.

[28] Del Prete G.Q., Keele B.F., Fode J. (2017). A single gp120 residue can affect HIV-1 tropism in macaques. PLoS Pathog.

[29] Swanstrom A.E., Immonen T.T., Oswald K. (2021). Antibody-mediated depletion of viral reservoirs is limited in SIV-infected macaques treated early with antiretroviral therapy. J Clin Invest.

[30] Del Prete G.Q., Smedley J., Macallister R. (2016). Short communication: comparative evaluation of coformulated injectable combination antiretroviral therapy regimens in simian immunodeficiency virus-infected rhesus macaques. AIDS Res Hum Retroviruses.

[31] Cline A.N., Bess J.W., Piatak M., Lifson J.D. (2005). Highly sensitive SIV plasma viral load assay: practical considerations, realistic performance expectations, and application to reverse engineering of vaccines for AIDS. J Med Primatol.

[32] Simonetti F.R., Sobolewski M.D., Fyne E. (2016). Clonally expanded CD4+ T cells can produce infectious HIV-1 in vivo. Proc Natl Acad Sci U S A.

[33] Somsouk M., Dunham R.M., Cohen M. (2014). The immunologic effects of mesalamine in treated HIV-infected individuals with incomplete CD4+ T cell recovery: a randomized crossover trial. PLoS One.

[34] Thomas J.A., Gagliardi T.D., Alvord W.G., Lubomirski M., Bosche W.J., Gorelick R.J. (2006). Human immunodeficiency virus type 1 nucleocapsid zinc-finger mutations cause defects in reverse transcription and integration. Virology.

[35] Neyens M., Crauwels H.M., Perez-Ruixo J.J., Rossenu S. (2021). Population pharmacokinetics of the rilpivirine long-acting formulation after intramuscular dosing in healthy subjects and people living with HIV. J Antimicrob Chemother.

[36] Yanez J.A., Remsberg C.M., Sayre C.L., Forrest M.L., Davies N.M. (2011). Flip-flop pharmacokinetics--delivering a reversal of disposition: challenges and opportunities during drug development. Ther Deliv.

[37] Spanarello S., La Ferla T. (2014). The pharmacokinetics of long-acting antipsychotic medications. Curr Clin Pharmacol.

[38] Hodge D., Back D.J., Gibbons S., Khoo S.H., Marzolini C. (2021). Pharmacokinetics and drug-drug interactions of long-acting intramuscular cabotegravir and rilpivirine. Clin Pharmacokinet.

[39] Landovitz R.J., Donnell D., Tran H. (2022).

[40] Centers for Disease Control and Prevention Effectiveness of prevention strategies to reduce the risk of acquiring or transmitting HIV. https://www.cdc.gov/hiv/risk/estimates/preventionstrategies.html#anchor_1562942347.

[41] Hatziioannou T., Ambrose Z., Chung N.P. (2009). A macaque model of HIV-1 infection. Proc Natl Acad Sci U S A.

[42] Hansen S.G., Piatak M., Ventura A.B. (2013). Immune clearance of highly pathogenic SIV infection. Nature.

