# Supplementary material for: Long-acting lenacapavir protects macaques against intravenous challenge with simian-tropic HIV
Source: eBioMedicine. 2023 Aug 23;95:104764. doi: 10.1016/j.ebiom.2023.104764 (PMC10470178; doi:10.1016/j.ebiom.2023.104764)
Supplement: Supplementary Table S1 [file mmc1.docx]

| **Supplemental Table 1: Baseline Characteristics of Study Animals** | | | | | |
| --- | --- | --- | --- | --- | --- |
| Animal ID | Study Group | Sex | Age (y) | Weight (kg) | CD4 Count (cells/µl blood)^a^ |
| A15W042 | PK (15 mg/kg) | Male | 5.0 | 8.5 | N.D. |
| Z15332 | PK (15 mg/kg) | Male | 5.1 | 7.8 | N.D. |
| Z15341 | PK (15 mg/kg) | Male | 5.0 | 8.2 | N.D. |
| A15W041 | PK (15 mg/kg) | Male | 5.0 | 7.7 | N.D. |
| Z15403 | PK (15 mg/kg) | Male | 4.9 | 8.4 | N.D. |
| Z16072 | PK (15 mg/kg) | Male | 4.6 | 6.7 | N.D. |
| Z12177 | IV Challenge, Vehicle | Male | 8.5 | 12.3 | 409 |
| Z13091 | IV Challenge, Vehicle | Male | 7.8 | 13.7 | 391 |
| Z13191 | IV Challenge, Vehicle | Male | 7.5 | 15.3 | 1102 |
| Z13236 | IV Challenge, Vehicle | Male | 7.4 | 12.4 | 892 |
| Z12223 | IV Challenge, TDF/FTC/DTG | Male | 8.6 | 13.4 | 992 |
| Z13095 | IV Challenge, TDF/FTC/DTG | Male | 7.8 | 13.8 | 514 |
| Z15265 | IV Challenge, TDF/FTC/DTG | Male | 5.6 | 9.6 | 496 |
| A15W048 | IV Challenge, LEN | Male | 5.1 | 9.6 | 1316 |
| Z15257 | IV Challenge, LEN | Male | 5.5 | 9.6 | 728 |
| Z15267 | IV Challenge, LEN | Male | 5.5 | 12.1 | 967 |

^a^N.D., not done.
