# Supplementary material for: Long-acting lenacapavir protects macaques against intravenous challenge with simian-tropic HIV
Source: eBioMedicine. 2023 Aug 23;95:104764. doi: 10.1016/j.ebiom.2023.104764 (PMC10470178; doi:10.1016/j.ebiom.2023.104764)
Supplement: Supplementary Table S2 [file mmc2.docx]

| **Supplemental Table 2: Injection site reactions, cumulative incidence** | | | | | |
| --- | --- | --- | --- | --- | --- |
| **LEN Dose** | | **Nodule** | **Erythema** | **Hyperkeratosis** | **Ulceration** |
| 15 mg/kg | Dose 1 | 3/3 | 3/3 | 2/3 | 0/3 |
|  | Dose 2 | 1/3 | 0/3 | 0/3 | 0/3 |
| 25 mg/kg | Dose 1 | 3/3 | 0/3 | 2/3 | 2/3 |
| 50 mg/kg | Dose 1 | 3/3 | 0/3 | 0/3 | 1/3 |
|  | Dose 2 | 3/3 | 0/3 | 2/3 | 2/3 |
| Vehicle | Dose 1 | 0/4 | 0/4 | 4/4 | 4/4 |
